# Diabetes Remission After LRYGBP With and Without Fundus Resection: a Randomized Clinical Trial

**DOI:** 10.1007/s11695-023-06857-z

**Published:** 2023-10-02

**Authors:** Dimitrios Kehagias, Charalampos Lampropoulos, Neoklis Georgopoulos, Ioannis Habeos, Dimitra Kalavrizioti, Sotirios-Spyridon Vamvakas, Panagiota Davoulou, Ioannis Kehagias

**Affiliations:** 1 University of Patras, 26504 Rio, Greece; 2 Intensive Care Unit, Saint Andrew’s General Hospital, 26335 Patras, Greece; 3 Department of Obstetrics and Gynecology, Division of Reproductive Endocrinology, University of Patras Medical School, 26504 Rio, Greece; 4 Department of Internal Medicine, Division of Endocrinology and Diabetes, University Hospital of Patras, 26504 Rio, Greece; 5 Department of Nephrology and Renal Transplantation, University Hospital of Patras, 26504 Rio, Greece; 6 Department of Surgery, Division of Bariatric and Metabolic Surgery, University Hospital of Patras, 26504 Rio, Greece

**Keywords:** Gastric fundus, Bariatric surgery, Ghrelin, Diabetes mellitus type 2, Gastrointestinal hormones

## Abstract

**Background:**

Glycemic control, after metabolic surgery, is achieved in two stages, initially with neuroendocrine alterations and in the long-term with sustainable weight loss. The resection of the gastric fundus, as the major site of ghrelin production, is probably related with optimized glucose regulation. The aim of the present study is to investigate whether the modification of laparoscopic Roux-en-Y gastric bypass (LRYGBP) with fundus resection offers superior glycemic control, compared to typical LRYGBP.

**Materials and Methods:**

Participants were 24 patients with body mass index (BMI) ≥40kg/m^2^ and type II diabetes mellitus (T2DM), who were randomly assigned to undergo LRYGBP and LRYGBP with fundus resection (LRYGBP+FR). Gastrointestinal (GI) hormones [ghrelin, glucagon-like-peptide-1 (GLP-1), peptide-YY (PYY)] and glycemic parameters (glucose, insulin, HbA1c, C-peptide, insulinogenic index, HOMA-IR) were measured preoperatively, at 6 and 12 months during an oral glucose tolerance test (OGTT).

**Results:**

Ninety-five percent of patients showed complete remission of T2DM after 12 months. LRYGBP+FR was not related with improved glycemic control, compared to LRYGBP. Ghrelin levels were not significantly reduced at 6 and 12 months after LRYGBP+FR. GLP-1 and PYY levels were remarkably increased postprandially in both groups at 6 and 12 months postoperatively (*p*<0.01). Patients who underwent LRYGBP+FR achieved a significantly lower BMI at 12 months in comparison to LRYGBP (*p*<0.05).

**Conclusion:**

Fundus resection is not associated with improved glycemic regulation, compared to typical LRYGBP and the significant decrease in BMI after LRYGBP+FR has to be further confirmed with longer follow-up.

**Graphical Abstract:**

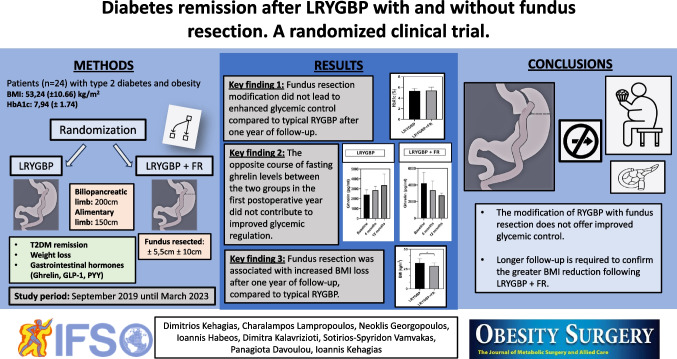

## Introduction

GI hormones and their changes in the immediate postoperative state appear to have a pivotal role in T2DM remission, creating a fertile field for research [1]. Particularly, GLP-1, PYY, and ghrelin are considered of the most well-studied gut hormones, while concomitantly mounting evidence exists demonstrating their implication in achieving glucose homeostasis [2].

From the aforementioned hormones, GLP-1 through the incretin effect is perhaps the most potent factor in T2DM remission, through increased response of pancreatic b-cells and amplified insulin secretion [3, 4]. In contrast, ghrelin, the only known orexigenic hormone, is produced predominantly from the gastric fundus and it appears from different preclinical and clinical studies to have a diabetogenic nature, by participating in glucose regulation with mechanisms that are not yet clearly elucidated [5, 6].

LRYGBP is considered one of the most effective bariatric procedures by balancing complication rate and metabolic benefit [7]. The resection of gastric fundus in conjunction with LRYGBP, as stated by different studies, is related with optimized antidiabetic effect [8, 9]. Furthermore, the decline in ghrelin levels after gastric fundus resection is probably implicated in early glycemic improvement independently of weight loss, suggesting a valuable glucoregulatory mechanism [10]. On the contrary, a number of studies reveal no differences in weight loss and glycemic control after gastric fundus resection, leading to controversy and obscurity regarding its exact role [11, 12].

In line with this notion, the modification of metabolic LRYGBP with fundus resection, in patients with obesity and T2DM, may appear as a metabolic weapon in the armamentarium of bariatric surgeon by ensuring optimized diabetic control through overwhelming activation of neuroendocrine mechanisms.

## Materials and Methods

### Trial Design

This was a prospective, non-blinded, randomized controlled trial (RCT), which took place in the Surgery Department of the University Hospital of Patras, in Greece from September 2019 until March 2023 and included patients with clinically severe obesity and T2DM. The trial was performed following the CONSORT guidelines. Based on the power analysis 24 patients were enrolled in the study, while a higher number of participants would increase the cost of the clinical trial. Furthermore, due to the COVID-19 pandemic, there was difficulty recruiting the above number of patients in a shorter time frame. Patients were randomly allocated (1:1) in LRYGBP and LRYGBP+FR, with the use of a random sequence generator software program. The trial was registered at clinicaltrials.gov (NCT05854875). All the patients were selected from the registry of the department of Metabolic and Bariatric Surgery and signed an informed consent. All procedures performed in this study involving human participants were in accordance with the ethical standards of the institutional research committee and with the 1964 Helsinki Declaration [13]. The study was approved by the local Research and Ethics Committee (No 730/10.12.2019). The primary outcome of the present study was HbA1c levels at one-year follow-up. The predefined secondary end-points included BMI, excess weight loss (EWL), glycemic parameters (glucose, C-peptide, insulin, insulinogenic index, HOMA-IR), and GI hormones (ghrelin, GLP-1, PYY).

### Participants

Twenty-four patients, aged 18 to 60 years old, with BMI ≥40kg/m^2^ and T2DM were included in the study. T2DM was defined according the criteria of the American Diabetes Association (ADA), as fasting glucose >126 mg/dl, impaired glucose values after 120-min oral glucose tolerance test 75gr (OGTT), HbA1c >6,5% or use of antidiabetic medications [14]. The duration of T2DM was determined less than 8 years, since longer duration is associated with irreversible b-cell function impairment and less recovery postoperatively [7]. A questionnaire was filled from each participant regarding eating habits, personal medical history and medications. Exclusion criteria were gestation, diabetes mellitus type I, alcohol or drug abuse, major depressive disorder, non-compliance with the medical personnel’s instructions, and previous abdominal surgeries with altered GI anatomy.

### Surgical Technique

All the operations were completed laparoscopically by the same surgeon at the surgical department of the University Hospital of Patras. Neither intraoperative complications nor conversion to open were reported. Both procedures included the creation of a small gastric pouch of 30ml capacity, a very long biliopancreatic limb of 200cm and an alimentary limb of 150cm. The aforementioned lengths are common practice for LRYGBP in our bariatric and metabolic surgery unit. The gastrojejunal anastomosis was created with a circular stapler of 25mm diameter after transoral placement of the anvil with assistance from the anesthesiologist. In the LRYGBP+FR group, the gastric body and fundus were mobilized by dividing the gastrocolic ligament and short gastric vessels until exposing the angle of His. After creating an L-shaped gastric pouch with the linear stapler, the fundus was resected with a horizontal transection at the level of the horizontal line of the gastric remnant. This selection was guided by previous experience with fundus resection modification in our department and relevant studies in the literature [8, 11]. A fundus with mean dimensions, ±5.5cm (width) and ±10cm (vertical length) was removed. The mean operative time was 18.1 ± 1,4 min longer in the LRYGBP+FR group.

### Blood Sampling and Laboratory Analysis

The participants were not allowed to drink or eat for 6 h prior to blood collection. Preoperatively antidiabetic medications were terminated for 48 h and GLP-1 analogues for one week. Blood sampling was carried out during an 75gr OGTT (0, 30, 60, 120min) preoperatively, at 6 and 12 months postoperatively.

Insulin was measured with enzyme-linked immunosorbent assay (ELISA) (Elecsys 2019, Roche) after serum collection. For the measurement of ghrelin, GLP-1 and PYY, blood collection was conducted with pre-cooled ethylenediaminetetraacetic acid (EDTA) vials, which contained 1,8 TIU (trypsin inhibitor units) of proteinase inhibitor, aprotinin (Trasylol). All the samples were centrifuged at 4°C for 20min, at 1600 RCF (Relative Centrifuge Force) and stored at −70°C until final analysis. For the above hormones analysis, commercial ELISA kits (Invitrogen, ThermoFisher Scientific) were utilized. All samples were collected, stored, and assayed at the same time in order to minimize the variation between groups.

### Statistical Analysis

Statistical analysis was provided by SPSS version 20.0 statistics software package (Statistical Package for the Social Sciences, SPSS Inc., Chicago, IL, USA). Curves and graphs were generated using GraphPad Prism version 5.0 (GraphPad Software, Inc., San Diego, CA). Sample size calculations were performed for mean HbA1c, in the first postoperative year, using a superiority design. Calculations were based on a test of the mean difference between LRYGBP and LRYGBP+FR, assuming a mean of 6.3 and a standard deviation of 1 in LRYGBP, according to previous findings in our department. An *α* level of .05 and power of 80% were used. The prespecified superiority margin for significant HbA1c reduction after LRYGBP+FR was a 20% greater decrease compared to LRYGBP. Based on these calculations, 10 patients per group were needed and considering a 20% dropout rate, a total of 24 patients were enrolled in the study. Continuous parameters were expressed as mean ± standard error (SE). The one-way analysis of variance (one-way ANOVA) was used for comparison of parameters for each type of surgery at different times (preoperatively, 6 months, 12 months).

In order to evaluate the interaction between different types of surgery (LRYGBP, LRYGBP+FR) and time (preoperatively, 6 months, 12 months), the two-way mixed ANOVA model was used. The same model was applied for evaluating the postprandial response (0,30,60,120 min) for each type of surgery at any time point (preoperatively, 6 months, 12 months).

For the comparisons of parameters and area under curve (AUC) levels, Mann-Whitney *U*-test was used. Statistical significance was defined as *p*<0.05. The correlation of different variables was evaluated with Kendall’s *τ* coefficient.

## Results

### Baseline Characteristics

**Fig. 1:**
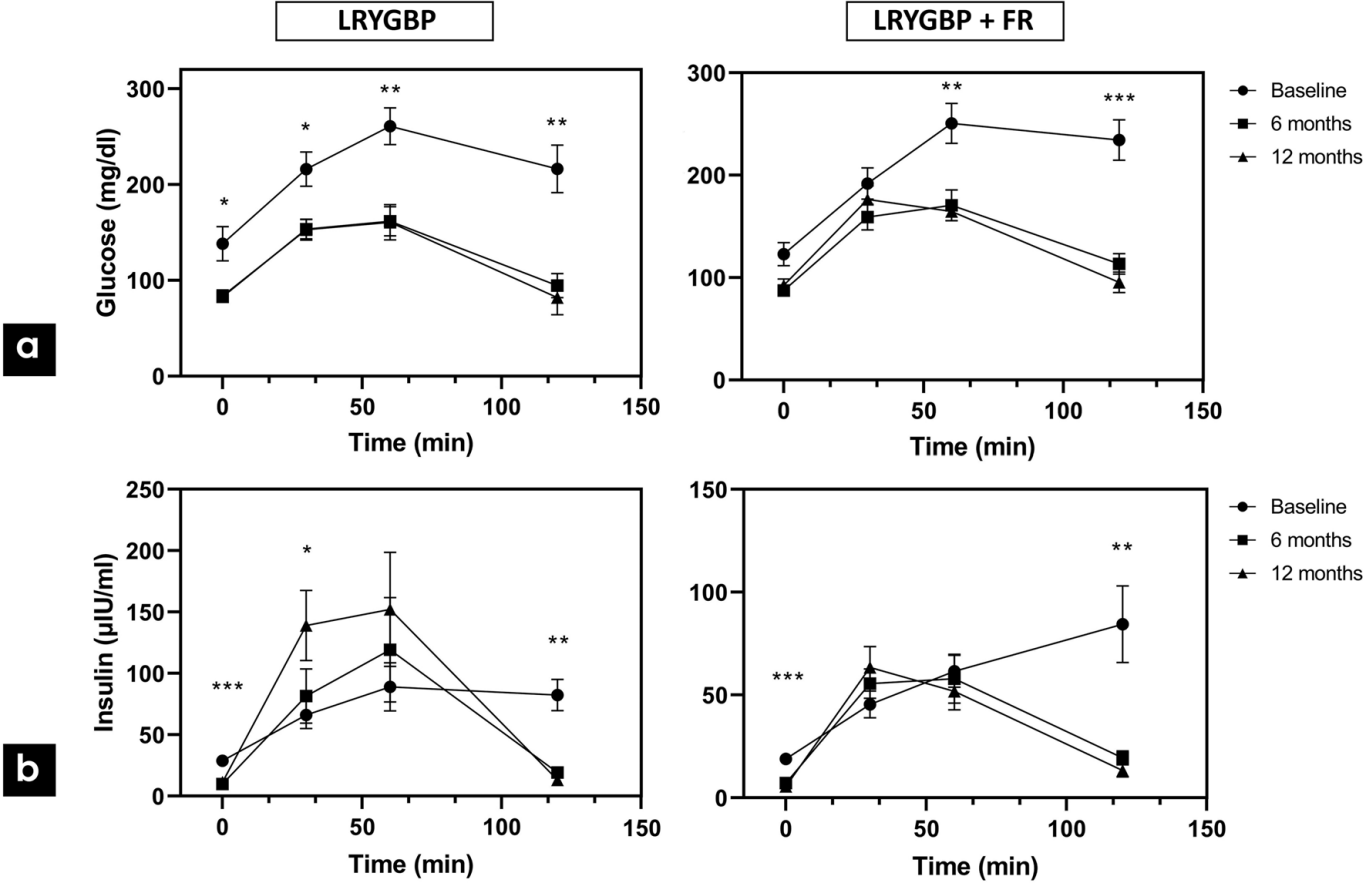
**a**, **b** Glucose and insulin secretion after each type of surgery, preoperatively, at 6 and 12 months *p<0.05, **p<0.01, ***p<0.001

**Fig. 2:**
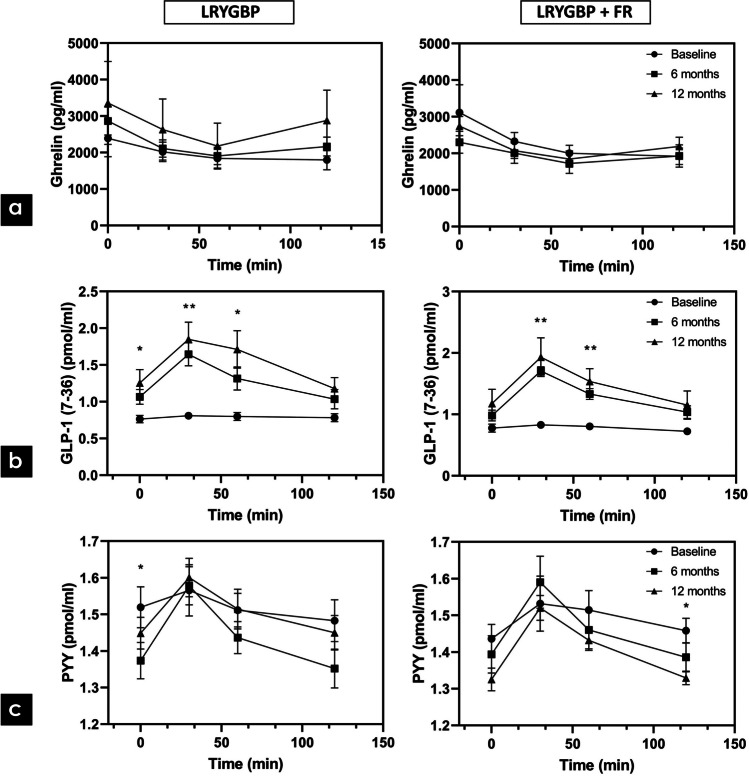
Gastrointestinal hormones secretion **a** ghrelin, **b** GLP-1, **c** PYY in each type of surgery preoperatively, at 6 and 12 months. *p<0.05, **p<0.01

**Fig. 3:**
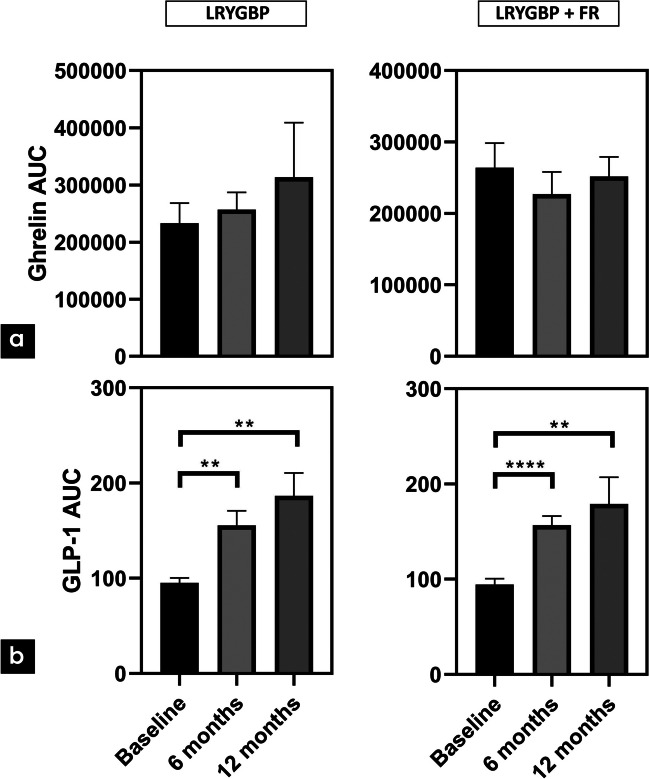
**a**, **b** Ghrelin and GLP-1 AUC levels at baseline, 6 months, 12 months after both types of surgery **p<0.01, ****p<0.0001

**Fig. 4:**
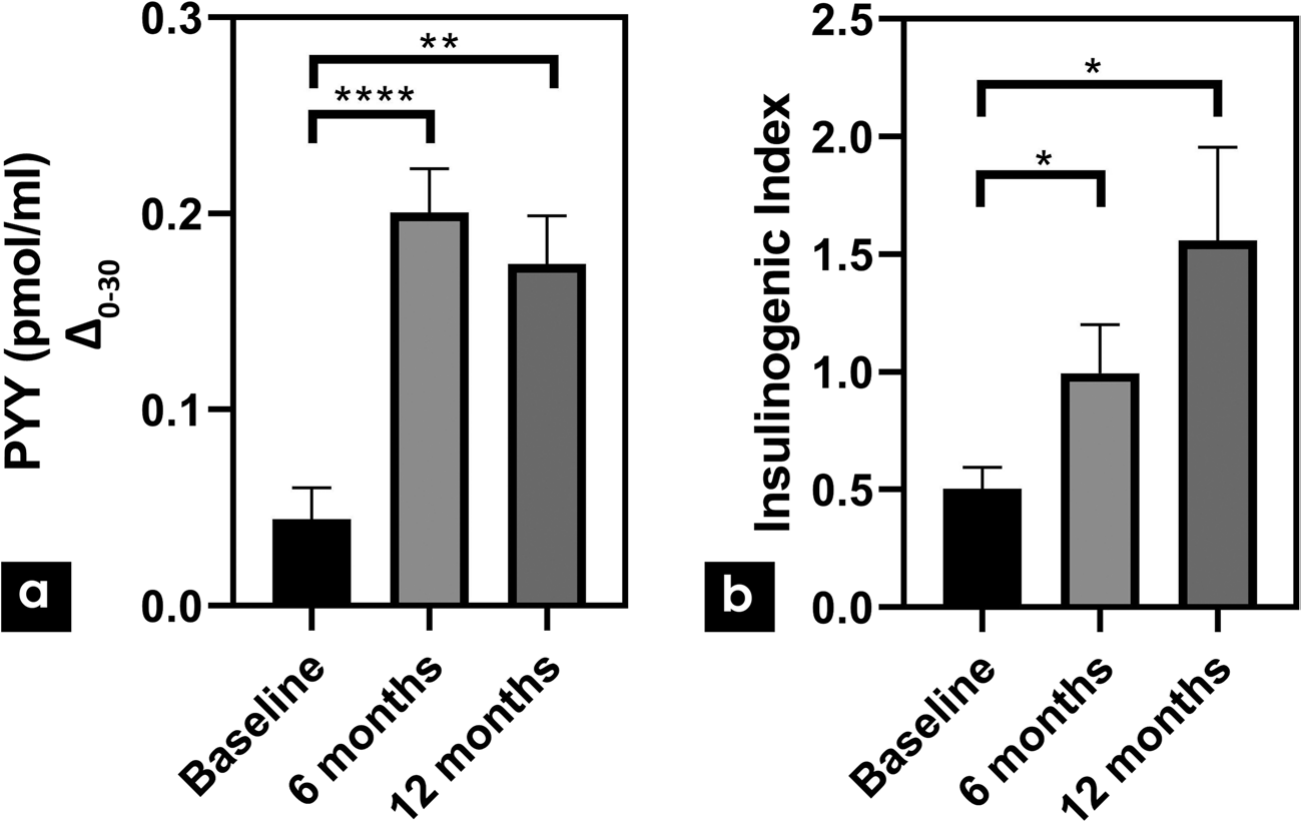
**a** PYY Δ_0-30_ levels in both types of surgery at baseline, 6 months and 12 months. **b** Insulinogenic index in both types of surgery *p<0.05, **p<0.01, ****p<0.0001

Between the two types of surgery, no statistically significant differences in BMI, age, and T2DM duration were observed. The patients had a mean (SD) age of 47 (±11) years and BMI 53 (±11) kg/m^2^. Among the 23 patients taking antidiabetic medications, 7 patients received insulin treatment and 4 used GLP-1 analogues (Table 1). One patient was newly diagnosed with T2DM and was not on any antidiabetic treatment. Concerning glycemic control, both groups were comparable to glycemic parameters (Table 2). In both types of surgery, impaired glucose tolerance was detected, with attenuated first and second phase insulin secretion and low insulinogenic index, without significant differences between them (Fig. 1). Furthermore, the two groups had similar fasting ghrelin, GLP-1 and PYY levels (Table 2). Ghrelin levels were decreased postprandially during the OGTT in both groups, without significant differences in AUC levels. GLP-1 postprandial secretion was blunted in both groups, with comparable AUC levels between them. Although a maximum postprandial secretion of PYY at 30 min was observed in both groups, these levels were not significantly higher compared to fasting levels. In both types of surgery, similar PYY AUC levels were observed preoperatively (Figs. 2, 3 and 4a).

**Table 1:** Baseline patient characteristics (mean ± SD)

Parameters	LRYGBP	LRYGBP+FR	*p*
Gender (M/F)	5/7	6/6	0.9992
Age (yr)	45.6 ± 10.7	49.9 ± 9.8	0.1234
BMI (kg/m^2^)	56.8 ± 11.5	49.7 ± 7.8	0.1727
Weight (kg)	166.2 ± 37.8	140.1 ± 28.9	0.0805
T2DM duration (yr)	3.20 ±1.95	4.33 ± 2.39	0.2397
HbA1c (%)	8.11 ± 1.66	7.78 ± 1.74	0.8315
HOMA-IR	10.79 ± 9.16	6.10 ± 3.80	0.1926
C-peptide (ng/ml)	4.87 ± 2.27	3.98 ± 1.15	0.2678
Anti-diabetic drugs (insulin) (GLP1 analogs)	11 (3) (1)	12 (4) (3)	

*p*<0.05 for significance

**Table 2:** Fasting values of glycemic parameters and gastrointestinal hormones in each group preoperatively, at 6 and 12 months after surgery (mean ± SD)

	LRYGBP				LRYGBP+FR			
		Mean ± SD	*p*_base_	*p*_6 months_	Mean ± SD	*p*_base_	*p*_6 months_	*p*_groups_
HbA1c (%)	Baseline	8.11 ± 1.66			7.78 ± 1.74			0.8315
	6 months	5.317 ± 0.480	**0.0030**		5.308 ± 0.598	**0.0008**		0.9435
	12 months	5.318 ± 0.447	**0.0042**	0.9999	5.417 ± 0.649	**0.0005**	0.9054	0.5748
C-peptide (ng/ml)	Baseline	4.87 ± 2.27			3.98 ± 1.15			0.2978
	6 months	3.025 ± 1.116	**0.0249**		2.533 ± 0.511	**0.0072**		0.2837
	12 months	2.491 ± 1.011	**0.0063**	0.2603	2.050 ± 0.485	**0.0009**	**0.0112**	0.2258
HOMA-IR	Baseline	10.79 ± 9.16			6.10 ± 3.80			0.1926
	6 months	2.411 ± 1.643	**0.0237**		1.336 ± 0.7004	**0.0037**		0.1005
	12 months	1.821 ± 1.345	**0.0438**	0.5791	1.276 ± 0.6025	**0.0022**	0.9546	0.3218
Glucose (mg/dl)	Baseline	138.2 ± 61.78			122.9 ± 39.10			0.5714
	6 months	82.75 ± 12.42	**0.0472**		87.42 ± 15.23	0.1032		0.9730
	12 months	83.64 ± 21.31	0.0893	0.9720	92.25 ± 21.79	0.0981	0.9933	0.9771
Insulin (μIU/ml)	Baseline	28.91 ± 16.56			18.925 ± 7.631			0.2893
	6 months	9.858 ± 7.512	**0.0004**		7.183 ± 3.069	**0.0005**		0.9999
	12 months	11.364 ± 9.979	0.0198	0.8975	5.500 ± 1.845	**0.0002**	0.2597	0.9992
GLP-1	Baseline	0.7636 ± 0.1721			0.7769 ± 0.2305			0.9999
	6 months	1.066 ± 0.3474	**0.0370**		0.9823 ± 0.3071	0.0535		0.7876
	12 months	1.256 ± 0.6056	**0.0247**	0.2811	1.176 ± 0.8064	0.1049	0.6393	0.9157
PYY	Baseline	1.519 ± 0.1937			1.436 ± 0.1367			0.4011
	6 months	1.374 ± 0.1727	**0.0454**		1.393 ± 0.1760	0.6103		0.8984
	12 months	1.449 ± 0.1436	0.6484	0.4067	1.325 ± 0.1067	0.1170	0.3485	**0.0149**
Ghrelin	Baseline	2388 ± 1747			4208 ± 4484			0.1277
	6 months	2864 ± 1327	0.6675		3369 ± 3817	0.7671		0.7987
	12 months	3359 ± 3773	0.7333	0.8881	2743 ± 910.4	0.4380	0.8009	0.5254

The numbers in bold is the statistical significance (*p* < 0.05)

*p*_base_ for comparison of baseline with any time, *p*_6months_ for comparison between 6 and 12 months, *p*_groups_ for comparison between each type of surgery

### Glycemic Control

All participants showed robust glycemic improvement and antidiabetic medications were discontinued, without need for recommencing at 6 months. Among 24 patients, 23 (95%) had complete remission of T2DM, while one patient from the LRYGBP+FR group experienced relapse of diabetes and reintroduction to oral medications at 12 months. HbA1c, C-peptide, and HOMA-IR demonstrated significant amelioration in both groups (*p*<0.05), not only at 6 months but also after 12 months, without differences between the two groups (Table 2). Fasting and postprandial glucose AUC values were similarly improved in both types of surgery at 6- and 12-months follow-up (Fig. 1a).

Fasting insulin values showed comparable decrease in both groups at 6 and 12 months (*p*<0.05). Insulin curves were alternated after both surgeries, with enhanced first phase insulin secretion and a shorter duration of the second phase. Insulin AUC levels were not significantly decreased after both types of surgery (Fig. 1b).

The insulinogenic index was increased statistically significant in both groups (*p*<0.05) (Fig. 4b).

### Weight Loss

BMI was remarkably reduced in both groups after 6 and 12 months of follow-up (*p*<0.0001). EWL was similar after each type of surgery (LRYGBP 66%, LRYGBP+FR 74%, *p*=0.228). Even though at 6 months after surgery, no difference in BMI decrease was observed (LRYGBP 37.86 ± 6.186, LRYGBP+FR 34.22 ± 5.594, *p*=0.0761), at 12 months, LRYGBP+FR demonstrated significant decrease in BMI compared to LRYGBP (LRYGBP+FR 28.88 ± 3.623 and LRYGBP 32.53 ± 5.387, *p*=0.0256) (Table 3).

**Table 3:** Excess weight loss (EWL%) and BMI changes for each type of surgery

	LRYGBP			LRYGBP+FR		
		Mean ± SD	*p*_base_	Mean ± SD	*p*_base_	*p*_groups_
% EWL	6 months	54.92 ± 6.551		54.92 ± 8.931		0.7012
	12 months	66.05 ± 12.50		74.27 ± 11.44		0.2288
BMI (kg/m^2^)	Baseline	56.78 ± 11.96		49.71 ± 8.198		0.1727
	6 months	37.86 ± 6.186	**< 0.0001**	34.22 ± 5.594	**< 0.0001**	0.0761
	12 months	32.53 ± 5.387	**< 0.0001**	28.88 ± 3.623	**< 0.0001**	**0.0256**

The numbers in bold is the statistical significance (*p* < 0.05)

*p*_base_ for the comparison with baseline values, *p*_groups_ for the comparison between groups

### Changes in Ghrelin Levels

Although LRYGBP+FR was associated with lower fasting ghrelin levels at 6 and 12 months, following LRYGBP, fasting ghrelin levels were progressively elevated at 6 and 12 months. However, these results did not manage to reach statistically significant levels (p>0.05). Postprandial AUC ghrelin levels were not significantly changed after both procedures. At 12 months, LRYGBP+FR indicated lower, albeit non-significant, AUC levels, compared to LRYGBP (Figs. 2a and 3a).

### Changes in GLP-1 Levels

Fasting GLP-1 levels were comparable between the two procedures postoperatively. Postprandially, both procedures resulted in significant GLP-1 secretion at 6 months and 12 months, with peak levels at 30 min during OGTT (*p*<0.01). The AUC levels were significantly increased in both groups (*p*<0.01), without any superiority between the two types of surgery (Figs. 2b and 3b).

### Changes in PYY Levels

Following LRYGBP, fasting PYY levels were significantly decreased at 6 months (*p*<0.05) and then progressively, at 12 months, returned to preoperative levels. However, after LRYGBP+FR fasting levels were significantly decreased at 12 months (*p*<0.05) (Table 2). PYY response to OGTT after 6 and 12 months was similar in both types of surgery, without any significant differences compared to preoperative levels (Fig. 2c). However, the difference in PYY levels from 0 to 30min (Δ_0-30_) as maximum postprandial secretion was statistically significant increased after both types of surgery at 6 (*p*<0.0001) and 12 months (*p*<0.01), in comparison to preoperative levels (Fig. 4a). No differences in AUC levels or Δ_0-30_ were observed between the two procedures.

### Correlations of Glycemic Parameters and Gut Hormones

BMI was strongly positively related to glycemic parameters (HbA1c, glucose, insulin) (*p*<0.01). On the other hand, a significant negative relationship was indicated with fasting GLP-1 levels (*p*<0,01) and less significant with fasting ghrelin levels (*p*<0.05). Fasting ghrelin levels were negatively correlated with fasting insulin levels (*p*<0.01). Fasting GLP-1 levels were strongly negative associated to glycemic parameters (HbA1c, glucose, insulin) (Table 4).

**Table 4:** Correlations between fasting levels of glycemic parameters and gastrointestinal hormones using the Kendall’s *τ* coefficient

		BMI	HbA1c	C-peptide	HOMA IR	Glucose	Insulin	Ghrelin	GLP-1
BMI	Correlation coefficient	1.000	.328^**^	.333^**^	.418^**^	.265^**^	.395^**^	−.165^*^	−.428^**^
	Sig. (2-tailed)		.000	.000	.000	.001	.000	.042	.000
	*N*	71	71	71	71	71	71	71	71
HbA1c	Correlation coefficient	.328^**^	1.000	.250^**^	.427^**^	.610^**^	.404^**^	−.066	−.353^**^
	Sig. (2-tailed)	.000		.003	.000	.000	.000	.424	.000
	*N*	71	71	71	71	71	71	71	71
Glucose	Correlation coefficient	.265^**^	.610^**^	.311^**^	.485^**^	1.000	.397^**^	−.121	−.292^**^
	Sig. (2-tailed)	.001	.000	.000	.000		.000	.139	.000
	*N*	71	71	71	71	71	71	71	71
Insulin	Correlation coefficient	.395^**^	.404^**^	.664^**^	.794^**^	.397^**^	1.000	−.252^**^	−.362^**^
	Sig. (2-tailed)	.000	.000	.000	.000	.000		.002	.000
	*N*	71	71	71	71	71	71	71	71
Ghrelin	Correlation coefficient	−.165^*^	−.066	−.158	−.183^*^	−.121	−.252^**^	1.000	−.049
	Sig. (2-tailed)	.042	.424	.054	.025	.139	.002		.545
	N	71	71	71	71	71	71	71	71
GLP-1	Correlation coefficient	−.428^**^	−.353^**^	−.346^**^	−.413^**^	−.292^**^	−.362^**^	−.049	1.000
	Sig. (2-tailed)	.000	.000	.000	.000	.000	.000	.545	
	*N*	71	71	71	71	71	71	71	71

**Correlation is significant at the 0.01 level (2-tailed) *correlation is significant at the 0.05 level (2-tailed)

## Discussion

In the present study, LRYGBP+FR did not elicit enhanced glycemic control and, interestingly, it was not related with a significant postoperative decrease in fasting ghrelin levels, compared to LRYGBP. Glycemic control in both types of surgery was attributed to successful weight loss and to remarkably increased postprandial secretion of GLP-1 and PYY. The first phase of insulin secretion and the second phase were exceptionally improved, demonstrating a progressive recovery of b-cell function and increased insulin sensitivity respectively. A noteworthy finding was the statistically significant decrease in BMI at 12 months following LRYGBP+FR. Nevertheless, this finding was not accompanied by significant changes in the studied neuroendocrine factors.

Ghrelin, the hormone of the gastric fundus, is an attractive target for obesity and T2DM, since it promotes insulin resistance and hyperglycemia [5, 15]. Obesity is characterized by lower fasting ghrelin levels, compared to lean subjects and attenuated postprandial suppression leading to reduced satiety and weight gain, implying a strong relationship with energy balance [16–18]. Insulin levels and HOMA-IR seem to be negatively related to total ghrelin, while insulin probably inhibits ghrelin secretion in lean and obese subjects [19, 20]. In our study, by using the Kendall rank correlation coefficient, a significant negative correlation was noticed between fasting ghrelin and insulin levels (*p*<0.01), suggesting indirectly the implication of ghrelin in glycemic control. Chronaiou et al. compared LRYGBP with LRYGBP+FR and observed significantly lower fasting ghrelin levels in the second group, although without including patients with T2DM [8]. Casajoana et al. studied the changes in GI hormones in patients with severe obesity and T2DM and, interestingly, an increase in fasting ghrelin levels was demonstrated 1 year after laparoscopic sleeve gastrectomy (LSG), considering this as a potential cause for diabetes relapse [21]. In the current study, fasting ghrelin levels did not show significant changes postoperatively between the two groups, probably due to the small sample size. However, after LRYGBP+FR, fasting ghrelin levels decreased at 6 and 12 months, while after LRYGBP, ghrelin levels followed the opposite course. This different fluctuation of fasting ghrelin levels between the two groups is an important finding, albeit not statistically significant. The resection of the gastric fundus, including the removal of the majority of ghrelin cells, is most probably the main culprit. On the other hand, the extent of fundus resection is another possible explanation for the non-significant decrease in ghrelin levels, since the exact size to be removed has not yet been determined. Ghrelin is also produced in other organs that may compensate for the loss after fundus resection, creating further controversies [22].

In this recent study, 95% of patients showed complete T2DM remission, which was attributed to different mechanisms according to the literature [3, 23]. Initially, EWL% (66% after LRYGBP and 74% after LRYGBP+FR) in the first postoperative year contributed to late glycemic control. However, in the early postoperative period, neuroendocrine mechanisms, elicited by altered gut anatomy, resulted in glycemic control, underpinning a two-step hypothesis [24, 25].

According to the hindgut theory, LRYGBP provokes increased GLP-1 and PYY secretion, through the transfer of nutrients to the distal intestine [26]. Based on the findings of this study, after both procedures, increased fasting and postprandial levels of GLP-1 were observed after 6 and 12 months. Moreover, bivariate analysis for fasting parameters showed that GLP-1 levels were strongly negatively related to HbA1c, glucose, and insulin, indicating its crucial role in glycemic control. The first phase of insulin secretion consists of a sharp increase lasting about 10 min, followed by the second phase which is a subsequent increase in insulin, depending on the stimulus duration [27, 28]. Both phases were improved postoperatively in the two groups, leading to recovered b-cell function and significant increase of insulinogenic index (*p*<0.05) [29].

From studies so far, it is known that PYY is not only an anorexigenic hormone, related to weight loss, but also is involved in the improvement and survival of b-cells [30, 31]. Based on the maximum postprandial secretion of PYY at 30min, we calculated the difference Δ_0-30_ postoperatively, which was significantly increased after both types of surgery at 6 and 12 months, suggesting another potential glucoregulatory mechanism. LRYGBP+FR was not related with an improved PYY secretion pattern, although Chronaiou et al. demonstrated significant postprandial PYY secretion after gastric fundus resection [8]. Other studies though, comparing LRYGBP with LSG, failed to achieve significant postprandial increased levels of PYY [32, 33].

Another important finding of this RCT was the statistically significant reduction in BMI after LRYGBP+FR, compared to LRYGBP. Chronaiou et al. with an RCT comparing these procedures did not show any changes in BMI, despite lower ghrelin levels in the fundus resection group [8]. Delko et al. performed a revision with fundus resection, in patients who had undergone LRYGBP with long biliopancreatic limb, without demonstrating any difference in BMI reduction [11]. Based on the findings of the current study, ghrelin levels were not significantly reduced after LRYGBP+FR, in order to justify the significant weight loss compared to LRYGBP. Searching for other potential factors, we noticed significantly decreased fasting PYY levels following LRYGBP+FR, which was a consequence and not a cause of weight loss [34]. Although this significant decrease in BMI was not clearly attributed to any studied mechanism, there are still more than a hundred peptides and other factors in the GI tract with yet unknown physiology and interaction between them [35].

Even though the current study led to some valuable conclusions, there are a number of limitations that should be taken into account. First and foremost, the small number of participants might be responsible for the non-significant differences by creating variation in the measured values. GI hormones were assayed after the collection of all samples; hence, the variability was difficult to predict in order to increase the number of studied patients during the trial. Lack of pilot data regarding GI hormones levels further contributed to the variability of the results. Therefore, the small sample size may lead to arbitrary conclusions and these results have to be cautiously interpreted. Although our study showed a significant difference in BMI after LRYGBP+FR, the mean BMI at baseline was marginally not significant in the fundus resection group (*p*=0.17). Nevertheless, this is an RCT that amplifies the significance of the results and challenges the mechanisms of glycemic control and weight loss after modification of LRYGBP with fundus resection. According to the data so far, more well-designed studies are needed to clearly elucidate the role of the gastric fundus in glycemic control and weight loss. Finally, a longer follow-up of these patients will confirm the significantly decreased BMI following LRYGBP+FR and whether this is preserved over time.

## Conclusion

The involvement of neuroendocrine mechanisms in glycemic control after bariatric surgery is an ongoing study subject. Based on the above findings, fundus resection is not associated with improved glycemic control, compared to typical LRYGBP and the significant decrease in BMI after LRYGBP+FR has to be further confirmed with longer follow-up. The exact mechanisms for the implication of gastric fundus in weight loss and glycemic control are still not clarified, requiring more well-designed studies targeting the above findings.

## Data Availability

The data that support the findings of this study are available on request from the corresponding author.
